# Unveiling Hydrogen-Based Direct Reduction Mechanisms of Multicomponent Oxides via *In Situ* High-Energy X‑ray Diffraction

**DOI:** 10.1021/acssuschemeng.5c12301

**Published:** 2026-01-19

**Authors:** Shiv Shankar, Barak Ratzker, Claudio Pistidda, Dierk Raabe, Yan Ma

**Affiliations:** † 28272 Max Planck Institute for Sustainable Materials, Max-Planck-Str. 1, 40237, Düsseldorf, Germany; ‡ Institute of Hydrogen Technology, 28338 Helmholtz-Zentrum hereon GmbH, D-21502 Geesthacht, Germany; § Department of Materials Science & Engineering, 2860 Delft University of Technology, Mekelweg 2, 2628 CD, Delft, The Netherlands

**Keywords:** *In situ* high-energy X-ray diffraction, Sintering, Hydrogen, Direct reduction, Sustainable metallurgy

## Abstract

Co-reduction of multicomponent oxides with hydrogen offers a carbon-neutral approach toward sustainable alloy design. Herein, we use *in situ* high-energy X-ray diffraction technique to gain insights into multicomponent oxide reduction of two precursor variants: mechanically mixed powders and pre-sintered oxide mixtures, targeting an equiatomic CoFeMnNi alloy. We find distinct reduction pathways and microstructure evolution, depending on initial precursors. Mixed powders are reduced to body-centered-cubic, face-centered-cubic, and MnO phases via halite, spinel, and Mn_3_O_4_ intermediates, whereas the pre-sintered complex oxide directly transforms into a mixture of metallic and MnO phases. The post-reduction microstructures were also strongly governed by the precursor state: mixed oxides exhibit loosely packed and coarse morphology, whereas the pre-sintered ceramic material showcases two distinct morphologies, either relatively dense metal-rich regions or regions with metallic nanoparticles supported on nanoporous MnO, highlighting the significant role of initial precursors on the final microstructure. Hence, precursor design strategies may offer a single-step route to nanoporous alloys with potential applications in catalysis and energy technologies.

## Graphical abstract

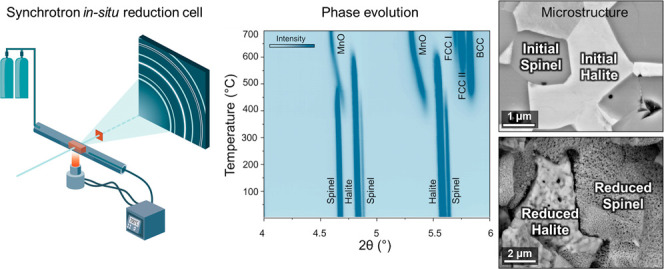

## 1 Introduction

Decarbonization of the metallurgical industry requires the development of sustainable routes for metal production, since current processes remain heavily reliant on fossil-based reductants.^1,2^ Traditional alloy synthesis is not only energy intensive, involving multiple high-temperature processes such as extraction, melting, homogenization, and casting, but also poses substantial strain on the environment as it contributes to nearly 40% of all industrial greenhouse gas emissions.^3^

Co-reduction of multicomponent oxides with hydrogen has therefore emerged as a sustainable approach to directly synthesize alloys with targeted applications.^4^ Various studies have investigated the hydrogen reduction behavior of mixed oxides, including binary,^5–9^ ternary,^10,11^ and multicomponent oxide systems.^12–14^ With increasing chemical complexity, the reduction mechanisms become more complex due to the different thermodynamic stabilities of the constituent metal oxides. In our previous study, we demonstrated that the initial precursor state, whether mechanically mixed powders or pre-sintered solid solution oxide mixtures, plays a decisive role in governing both the reduction pathway and the final microstructure.^15^ This is illustrated by the fact that, although both precursor types reached similar reduction degrees (∼80%) and primarily formed face-centered-cubic (FCC) and MnO phases, the pre-sintered sample exhibited an additional ∼ 1 wt. % body-centered-cubic (BCC) phase. This was attributed to the localized deficiencies of FCC-stabilizing elements (Co and Ni) in proximity to Fe that partitioned out of the (Fe,Mn)O solid solution. Hence, differences in the precursor states not only influence the corresponding chemical driving forces when exposed to co-reduction conditions but also the reduction pathways. Decoding the multiple reduction steps, particularly the formation of metastable intermediate phases is critical for understanding the hydrogen-based direct reduction (HyDR) mechanisms of multicomponent oxides. Furthermore, this knowledge provides a rational design guideline for one-step alloy production via the HyDR of multicomponent oxides.

Beyond precursor effects, co-reduction is also influenced by HyDR processing parameters, such as hydrogen partial pressure,^16,17^ temperature,^18^ holding time,^19^ and heating rate.^20^ The reduction temperature plays a crucial role in providing the thermodynamic driving force required for the reduction of metal oxides. In addition to thermodynamic constraints, the reduction process is also governed by kinetic factors. The co-reduction of multicomponent oxides is accompanied by reactive sintering of the newly formed metallic phases, which can lead to pore closure and progressively limit hydrogen access to the remaining unreduced oxides.^5,21–23^ Despite advances in understanding the reduction behavior of mixed oxides, most studies^24–26^ relied on thermogravimetric analysis and post-mortem microstructure characterization, failing to capture the real-time transient intermediate phases governing the underlying reduction mechanisms and microstructure evolution.

Therefore, here we studied the HyDR process using *in situ* synchrotron high-energy X-ray diffraction (HEXRD) of two precursor oxide mixtures: mechanically mixed powders and chemically mixed pre-sintered oxide mixtures. The *in situ* reduction measurements provided mechanistic insights into multicomponent oxide reduction by monitoring the reduction sequence and the transient phases involved in the reduction process. Microstructure characterization revealed distinctly different behavior: mixed powder reduced to a loosely packed metallic and oxide phase, whereas the pre-sintered sample developed a dual phase microstructure, comprising metallic nanoparticles supported on a nanoporous MnO matrix.

## 2 Experimental Section

### 2.1 Oxide Mixtures Preparation

To obtain the oxide precursors, four metal oxide powders, namely, Co_3_O_4_, Fe_2_O_3_, Mn_2_O_3_, and NiO, were mixed with a targeted equiatomic metallic concentration (25 at. % each). The powders were mechanically mixed and homogenized using a planetary ball mill (Fritsch 7). Some of the mixed powder samples were also subsequently compacted and sintered in an Ar atmosphere at 1100 °C to obtain a chemically blended pre-sintered sample consisting of Co,Ni-rich halite and Mn,Fe-rich spinel. Detailed sample preparation procedure and characterization of initial precursors can be found elsewhere.^15^ A rectangular prism specimen (∼0.5 × 0.5 × 2 mm^3^) was cut from the sintered sample using a diamond wire saw to fit into a sapphire capillary (0.6 mm inner diameter) used for the *in situ* HEXRD measurement. The mixed powder was manually compressed into a green body inside the capillary with the help of copper wires from both ends.

### 2.2 *In Situ* Hydrogen-Based Direct Reduction

The *in situ* synchrotron HEXRD reduction experiments were performed at 700 °C in hydrogen atmosphere using a capillary cell,^27^ as illustrated schematically in Figure 1. The sapphire capillary cell has an inner diameter of 0.6 mm and a wall thickness of 0.1 mm, chosen to ensure mechanical stability and minimize X-ray attenuation for 60 keV (λ = 0.207381 Å) X-rays. The attenuation by the capillary for the given X-ray energy is negligible (*I*/*I*_0_ > 0.98) and does not affect the phase identification or relative peak intensities. The measurements were conducted at the Powder Diffraction and Total Scattering Beamline, P02.1 of PETRA III in the Deutsches Elektronen-Synchrotron (DESY).^28^ Samples were placed between the incident beam and a Varex XRpad 4343CT fast area detector (2880 × 2880 pixels) with a sample-to-detector distance of ∼1700 mm and a beam size of 0.5 × 0.5 mm^2^. Debye–Scherrer diffraction rings were continuously recorded with an exposure time of 5 s. The capillary cell was heated by a ceramic resistive heater located beneath the capillary, and the sample temperature was measured by a type K thermocouple, as shown by Figure 2d inset. After the cell was flushed with Ar for 5 min, H_2_ (99.999%) was introduced at 2 bar total pressure from the gas inlet. The samples were heated to 700 °C with a ramping rate of 10 °C/min, held isothermally at 700 °C for 30 min, and then cooled down to room temperature within 15 min. A reduction temperature of 700 °C was selected because it provides sufficient thermodynamic driving force for the complete reduction of Fe, Co, and Ni oxides using hydrogen, while avoiding the excessively high temperatures (1200–1400 °C) required for MnO reduction.^29^ This temperature therefore represents a technologically relevant process window that enables both systematic investigation of precursor-dependent reduction pathways and intermediate phase formation.

**Figure 1:**
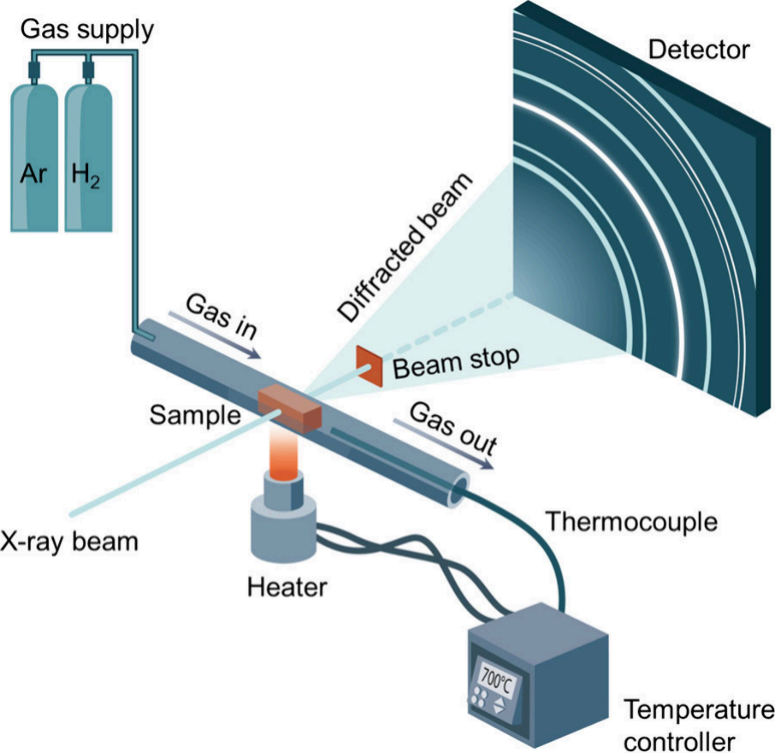
Schematic illustration of the *in situ* synchrotron high-energy X-ray diffraction (HEXRD) experimental setup coupled with a capillary cell and a ceramic resistive heater to investigate the reduction behavior of multicomponent oxides in a hydrogen atmosphere.

**Figure 2:**
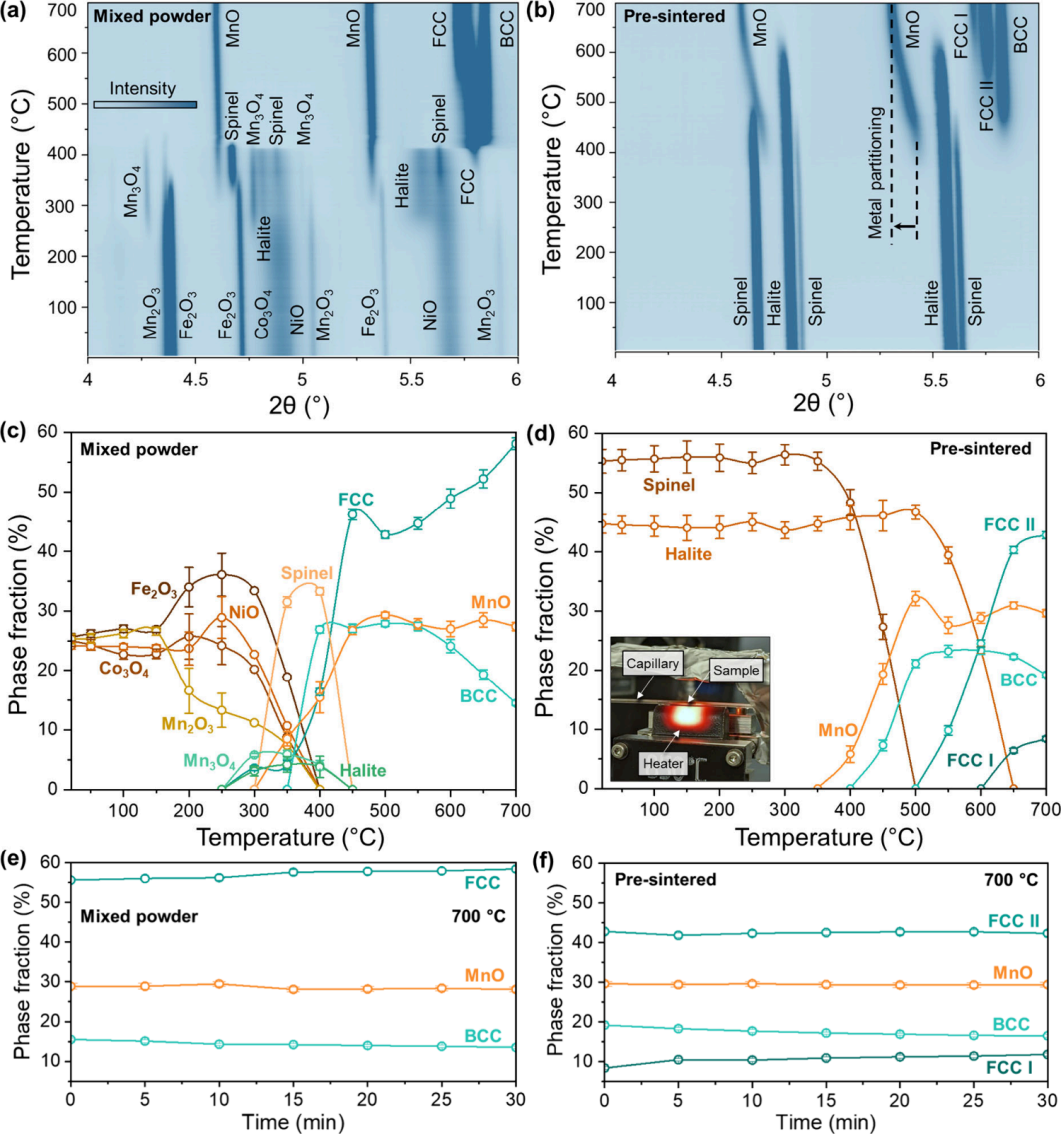
Contour maps of the *in situ* synchrotron high-energy X-ray diffraction (HEXRD) peak intensity as a function of temperature during heating in a hydrogen atmosphere to 700 °C with a ramping rate of 10 °C/min for (a) mixed powder and (b) pre-sintered samples. The plots in (c) and (d) show the corresponding phase fractions during heating up to 700 °C; (e, f) during isothermal holding at 700 °C. The inset in (d) presents a photograph of the *in situ* capillary cell setup.

Diffraction patterns were integrated using the general structure analysis system (GSAS-II) software.^30^ Phase identification was performed using the PDF-5+ database,^31^ and quantitative phase analysis was carried out by Rietveld refinement with a pseudo-Voigt function using the MDI JADE 10 software package.

### 2.3 Materials Characterizations

Microstructural characterization of the samples before and after reduction was conducted using a ZEISS Sigma 500 high-resolution scanning electron microscope (SEM) equipped with an EDAX APEX Advanced X-ray dispersive spectroscopy (EDS) detector, operated at an acceleration voltage and beam current of 15 kV and 7.7 nA, respectively.

## 3 Results and Discussion

### 3.1 Hydrogen Reduction of Mixed Powder and Pre-sintered Samples

The phase evolution during HyDR for the mixed powder and pre-sintered samples is shown in Figure 2. Prior to HyDR, the mixed powder sample exhibits diffraction patterns corresponding to individual metal oxides (Fe_2_O_3_, Co_3_O_4_, NiO, and Mn_2_O_3_), as shown in Figure 2a. Upon heating to 250–420 °C, the reduction proceeds sequentially through transient intermediate phases, including Mn_3_O_4_ (tetragonal spinel), spinel, and halite. With further heating up to 700 °C, the intermediate phases are reduced to FCC, BCC, and MnO. The observed formation of transient spinel and halite phases followed by their reduction to metallic and MnO phases agrees with prior thermodynamic prediction.^15^

In contrast, the pre-sintered sample initially consists of two phases: 55.3 ± 2.1 wt. % spinel and 44.7 ± 1.6 wt. % halite, as shown in Figure 2d. No reduction took place in the pre-sintered complex oxide sample until ∼350 °C, as the phase composition remained unchanged below this temperature. Upon further heating to ∼600 °C, the spinel phase reduces to a Mn,Fe-rich halite, where metallic Fe (as well as Co and Ni) partitions out of the MnO matrix and forms metallic solid solutions, as confirmed by the MnO peaks shifting to lower 2θ and concurrent emergence of BCC and FCC phases when heated up to 700 °C (Figure 2b). Both samples exhibit a continuous minor shift in all peaks to lower 2θ values during heating due to thermal lattice expansion. Beyond ∼400 °C, the negligible additional shift observed for the mixed powder (4.60° to 4.59°) indicates that thermal expansion contributes to a minor increase in lattice parameter, while in the pre-sintered sample, the order of magnitude larger shift (from 5.42° to 5.30°) reflects the reduction process and elemental partitioning of metals out of the halite phase expanding the MnO lattice.^32^

A similar reduction pathway is observed for the Co,Ni-rich halite phase, which completely reduces to the FCC phase. Notably, the halite phase is stable until ∼600 °C and has relatively higher thermodynamic stability compared with their individual oxide counterparts, i.e., Co_3_O_4_ and NiO, as previously shown by the multicomponent Ellingham diagram.^15^ Moreover, the *in situ* HEXRD data revealed two FCC variants, designated as FCC I (2θ_111_ = 5.69°) and FCC II (2θ_111_ = 5.76°). This is due to the difference in elemental concentrations of Fe, Co, and Ni in these FCC phases. Based on their lattice parameters (a), it can be concluded that FCC I (a = 3.613 Å) and FCC II (a = 3.574 Å) are Fe-rich and Fe-deficient phases, respectively, due to the larger metallic atomic radius of Fe compared with Co and Ni.^33^ Additionally, the onset temperature of BCC phase formation (∼470 °C) is lower than those of FCC I (∼560 °C) and FCC II (∼640 °C). Since the BCC and FCC phase primarily originates from Fe-rich, and Co, Ni-rich oxides, indicating that the intrinsic stability of chemically mixed multicomponent oxides does not follow the thermodynamic stability trend predicted by the Ellingham diagram.^34^ Thus, it can be concluded that the observed difference in reduction mechanisms arises due to the interplay between thermodynamics (phase stabilities), kinetics (gas–solid interactions and diffusion pathways), and morphological differences in the two precursor types.

The phase fractions estimated during heating to 700 °C for both samples are shown in Figure 2c and 2d. For the mixed oxide prior to HyDR, the individual metal oxides exhibit phase fractions of 25 ± 0.9 wt. %, confirming the targeted elemental concentration in the initial precursors (Figure 2c). As reduction progresses with increasing temperature up to 400 °C, the oxides intermix and undergo reactive sintering, resulting in the formation of 33.3 ± 0.6 wt. % spinel, 4.1 ± 0.8 wt. % Mn_3_O_4_, and 3.8 ± 1.8 wt. % halite. These intermediate oxides ultimately reduce to a mixture of 58.1 ± 1.0 wt. % FCC, 27.4 ± 0.7 wt. % MnO, and 14.5 ± 0.4 wt. % BCC. In contrast, the pre-sintered sample initially consists of 55.3 ± 2.0 wt. % spinel and 44.7 ± 1.6 wt. % halite, as shown in Figure 2d. Upon heating to 700 °C, these solid solution oxides yield 29.6 ± 0.6 wt. % MnO, 19.2 ± 0.4 wt. % BCC, 8.4 ± 0.4 wt. % FCC I (Fe-rich), and 42.8 ± 0.6 wt. % FCC II (Fe-deficient) phases. The phase fraction variation for mixed powder and pre-sintered samples during isothermal holding at 700 °C is shown in Figure 2e and 2f, respectively. For both precursor types, the MnO phase fraction (∼29.5 wt. %) remains nearly constant during the isothermal holding period, indicating that no further reduction occurs at this temperature. In contrast, the metallic phases exhibit some time-dependent phase evolution. For the mixed powder sample, the BCC phase fraction decreases from 15.5 ± 0.4 to 13.5 ± 0.3 wt. %, while the FCC phase fraction increases from 55.6 ± 0.8 to 58.4 ± 0.7 wt. % after holding at 700 °C for 30 min, indicating diffusion-driven mixing that increases the FCC phase fraction. Similarly, for the pre-sintered sample, the BCC phase fraction decreases from 19.2 ± 0.4 to 16.5 ± 0.4 wt. %, accompanied by an increase in the Fe-rich FCC I phase from 8.4 ± 0.4 to 11.8 ± 0.4 wt. %, while the FCC II (Fe-deficient) phase fraction remains nearly constant. Notably, the substantially higher fraction of the BCC phase compared to our previous study (∼1 wt. %) highlights the influence of non-equilibrium reduction conditions on the resulting phase partitioning.^15^ These trends indicate that isothermal holding enables further intermixing of the metallic phases via the kinetics that are much slower than during reduction.

Although the mixed oxide powders react and form intermediate spinel and halite phases during reduction (∼300–450 °C), the sequential reduction steps and phase transformation kinetics are different as is the final phase composition. Notably, the onset of MnO and BCC formation occurs at similar temperatures in the mixed powder (∼300–350 °C) and pre-sintered material (∼350–400 °C). The minor differences can be attributed to the reduction kinetics of powder versus bulk material, which are even lower for small specimens reduced in a capillary cell compared with larger samples reduced in conventional setups.^15^ Nevertheless, the FCC metallic phase forms at a significantly lower temperature in the mixed powder (∼250 °C) while at ∼ 400 °C for the pre-sintered sample. This correlates with the enhanced thermodynamic stability of the Co,Ni-rich halite phase in the pre-sintered sample. These results demonstrate that the initial precursor critically influences both the reduction pathways and the final phases, owing to the distinct phase composition and altered thermodynamic stability upon sintering prior to HyDR.^15^

### 3.2 Post Reduction Microstructure Characterization

Microstructures of mixed powder and pre-sintered samples before and after HyDR are presented in Figure 3. The mixed powder sample exhibits a porous compact particulate morphology (Figure 3a and 3b) with a homogeneous distribution of fine Co-, Fe-, and Ni-oxide particles (∼0.05–1 μm) and somewhat larger Mn oxide particles (∼0.2–6 μm). Conversely, the initial microstructure of the pre-sintered sample is a dense ceramic with a dual-phase morphology, consisting of Mn,Fe-rich spinel and Co,Ni-rich halite (Figure 3c and 3d). Note that after reduction the powder sample retained a cylindrical shape due to the prior compaction of the powders within the circular capillary cell (Figure 3e), while the pre-sintered sample retained its rectangular shape (Figure 3i). The morphology of the reduced mixed powder resembles that of the initial mixed powder sample, showing homogeneously distributed partially sintered agglomerates (Figure 3f-h). In contrast, the reduced pre-sintered sample exhibits a microstructure comprising two distinct morphologies closely resembling the initial dual-phase microstructure (Figure 3j and 3k). These two distinct morphological types reflect substantial differences in the microstructure evolution of the spinel and halite phases (Figure 3i-l). The reduced halite mostly metallic region is relatively dense (∼94%) with larger isolated pores (∼200 nm), whereas the reduced spinel region is highly porous (∼20%) and contains much finer pores (∼50 nm), owing to the predominant presence of unreduced MnO which inhibits sintering.^35^

**Figure 3:**
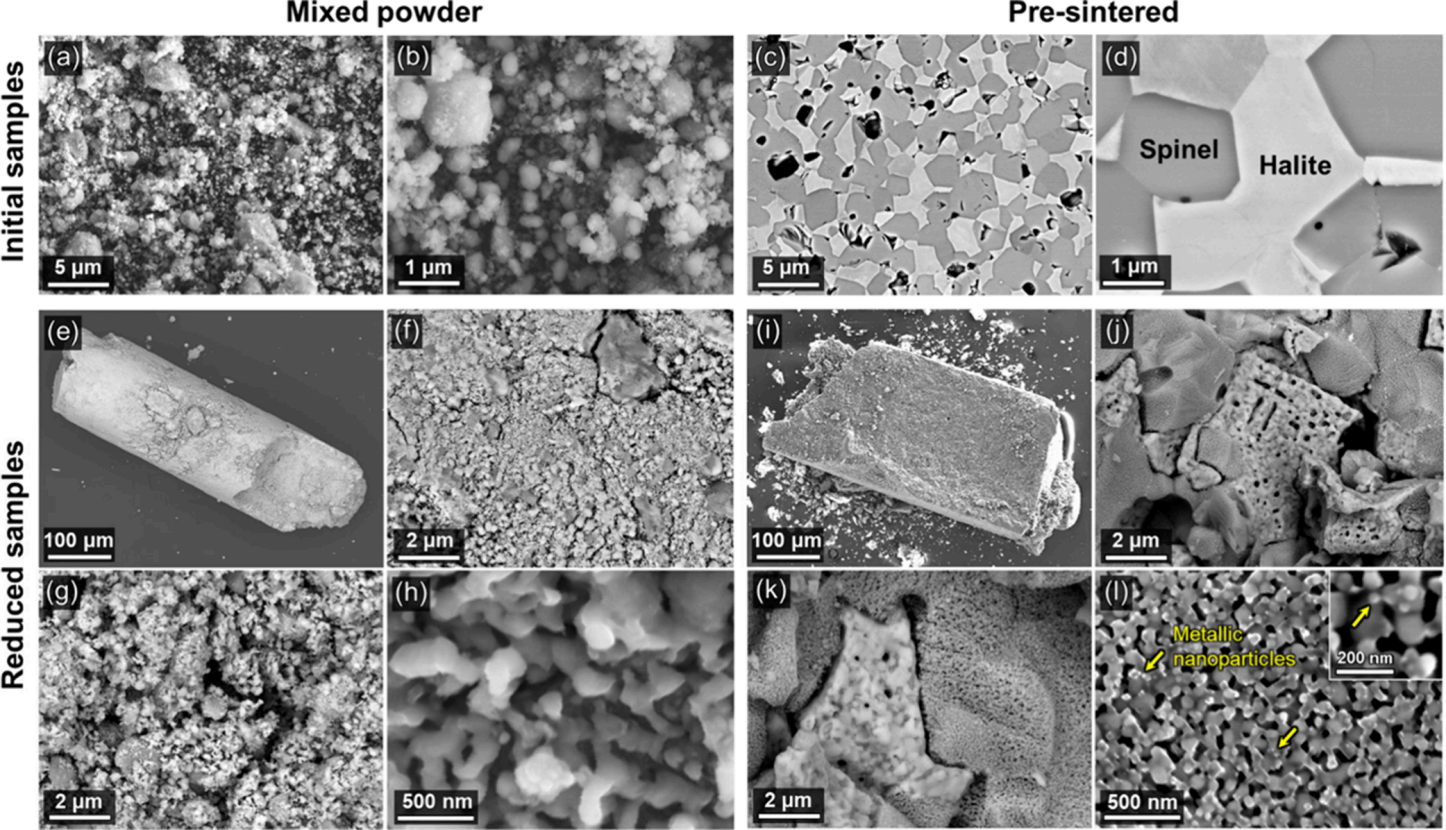
SEM micrographs of the precursors before reduction: (a, b) Mixed powder and (c, d) pre-sintered samples. SEM micrographs of the (e-h) mixed powder and (i-l) pre-sintered samples reduced in a hydrogen atmosphere at 700 °C, with a heating rate of 10 °C/min. The region in (l) corresponds to the nanoporous regions formed from the initial spinel phase. The arrows point to some of the metallic nanoparticles formed on the surface of a nanoporous oxide matrix.

High-magnification SEM micrographs reveal the formation of metallic nanoparticles (∼10–50 nm diameter) dispersed throughout the oxide matrix (Figure 3l), a phenomenon generally referred to as “exsolution”.^36^ In this process, a less stable metal oxide (i.e., Fe oxide) is initially in solid solution with a relatively highly stable metal oxide (i.e., Mn oxide). Under a reducing atmosphere, the less stable metal oxide metallizes by partitioning out of the oxide matrix and reduces to form metallic regions on the surface of the stable oxide. The development of nanoporous MnO is linked to the generation of fine pore networks during direct reduction.^37–39^ The reasons behind this microstructural evolution may lie in the combination of reducing small specimens (∼0.3–0.4 mm) and the reducing conditions (e.g., a high local hydrogen partial pressure) in the present case. This feature introduces the possibility of such materials as a supported catalyst for heterogeneous catalysis, where metallic nanoparticles (<20 nm) act as active sites.^40^ Additionally, the nanoporous oxide support can enhance catalytic activity by providing a higher surface area, improved mass transport, and increased accessibility of reactants to the active sites.^41^

EDS analysis of the reduced mixed powder and pre-sintered samples is presented in Figure 4. The elemental maps for the mixed powder reveal that the Mn signal corresponds to oxygen, confirming the presence of unreduced MnO (Figure 4b and 4d), along with the formation of an Fe, Co, and Ni-rich metallic phase, as shown by (Figure 4c, 4e, and 4f). Similarly, the pre-sintered sample shows formation of metallic Fe, Co, Ni and unreduced Mn oxide phases (Figure 4h-l). Particularly, the denser regions originated from the Co,Ni-rich halite were reduced to a metallic alloy consisting predominantly of Co and Ni with some Fe and embedded MnO, whereas the other regions originating from reduction of the Mn,Fe-rich spinel, involving the exsolution of metal from the stable MnO matrix and resulting in a nanoporous oxide skeleton peppered with abundant metallic nanoparticles. This transformation is evident from the substantial MnO peak shift above 400 °C during the *in situ* phase evolution of the pre-sintered sample (Figure 2b). The exsolution process during HyDR can be further controlled by adjusting the reducing conditions, such as hydrogen partial pressure and temperature.^42^

**Figure 4:**
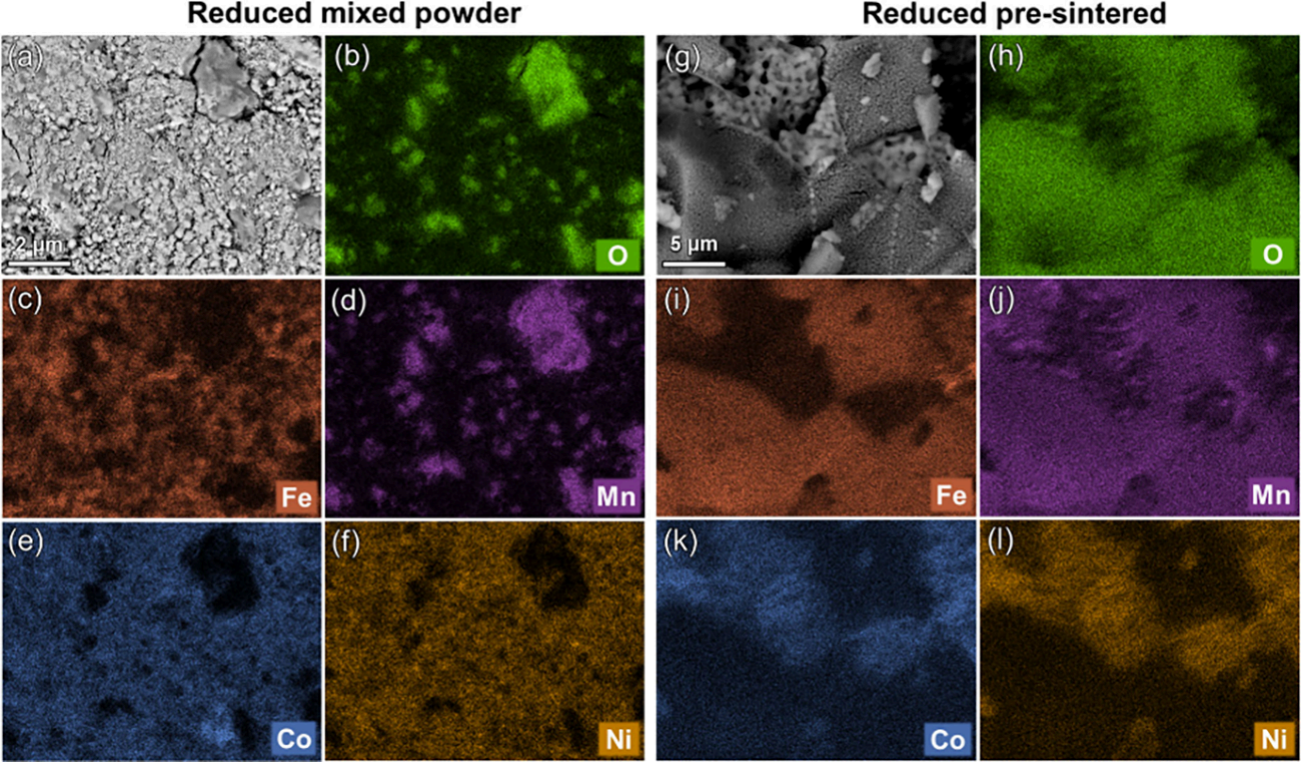
(a) SEM micrograph of the reduced mixed powder and corresponding EDS elemental maps of (b) O, (c) Fe, (d) Mn, (e) Co, and (f) Ni. (g) SEM micrograph of the reduced pre-sintered sample and corresponding EDS elemental maps of (h) O, (i) Fe, (j) Mn, (k) Co, and (l) Ni.

### 3.3 Elemental Partitioning during Hydrogen-Based Direct Reduction

Local EDS analysis was further performed to better understand the elemental intermixing and partitioning that took place during the HyDR of pre-sintered sample. EDS point scans were performed within the originally spinel ‘grain interior’ (spot 1) and at the ‘grain boundary’ (spot 2), as shown by the marked regions in Figure 5a. It was found that although the regions consist mostly of Fe and MnO, there are also considerable amounts of Co and Ni as well (see the EDS spectra and elemental fractions in Figure 5b). The metallic phase in the ‘grain interior’ consists of roughly 36 at. % Fe, 12 at. % Co, and 8 at. % Ni. Notably, the ‘grain boundaries’ are more enriched with Co and Ni on the expense of MnO, with roughly 35 at. % Fe, 15.7% at. Co, and 14 at. % Ni. This result suggests that during the HyDR process there was segregation of Co and Ni to the spinel grain boundaries, where a larger fraction of them exsolved. The presence of Co and Ni in the formerly spinel regions (and Fe and MnO in the halite regions) indicates substantial interdiffusion between the different solid solution phases during hydrogen reduction.

**Figure 5:**
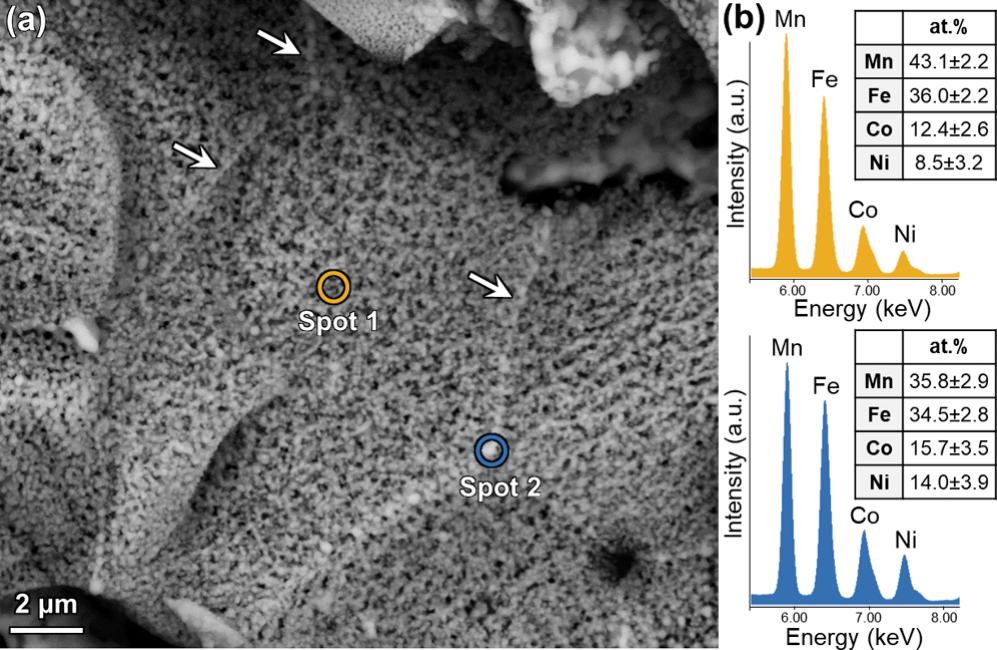
(a) SEM micrograph of Mn,Fe-rich region with spot 1 and spot 2 indicating the regions of formerly Mn,Fe-rich spinel ‘grain interior’ and ‘grain boundary’ examined by EDS point analysis, some ‘grain boundaries’ are marked with white arrows. (b) EDS spectra and quantitative results of Mn, Fe, Co, and Ni measured at spot 1 (top) and spot 2 (bottom), highlighting Co and Ni enrichment at regions corresponding to the former spinel grain boundaries.

## 4 Conclusions

Hydrogen-based direct reduction (HyDR) of mechanically mixed powder and chemically mixed pre-sintered multicomponent oxide mixtures was investigated via *in situ* high-energy X-ray diffraction (HEXRD) at 700 °C. The *in situ* HEXRD enabled direct tracking of the phase evolution during HyDR in real time, thereby providing mechanistic insight into interdiffusion, reactions, and reduction pathways in multicomponent oxide systems. Both precursor types were reduced to a mixture of metallic and oxide phases, yet via distinctively different reduction routes. The mixed powder reduced to FCC, BCC, and MnO through the formation of transient halite, spinel, and Mn_3_O_4_ phases, whereas the pre-sintered sample that contained Co,Ni-rich halite and Mn,Fe-rich spinel directly reduced to BCC, MnO, and two FCC (Fe-rich and Fe-deficient) phases. SEM analysis revealed distinctively different microstructures following hydrogen reduction of the two precursor types. The mixed powder sample exhibited loosely packed morphology, while the pre-sintered sample developed two distinct regions: a dense mostly metallic region rich in Co and Ni and a porous microstructure comprising Fe-rich metallic particles supported on a nanoporous MnO matrix. The results of this study emphasize that the HyDR of multicomponent oxides is highly dependent on the initial precursor state, dramatically influencing the phase and microstructure evolution. These can be further tailored by optimizing precursor selection, particle size, and elemental ratios as well as by modifying the HyDR conditions. Thus, this offers a promising pathway toward designing sustainable porous alloys in a single-step process with tunable functionalities.
